# Development of Therapeutic Approaches for Myotonic Dystrophies Type 1 and Type 2

**DOI:** 10.3390/ijms231810491

**Published:** 2022-09-10

**Authors:** Lubov Timchenko

**Affiliations:** Departments of Neurology and Pediatrics, Cincinnati Children’s Hospital Medical Center, the University of Cincinnati, Cincinnati, OH 45229, USA; lubov.timchenko@cchmc.org

**Keywords:** myotonic dystrophy, congenital myotonic dystrophy, myotonic dystrophy type 2, clinical trials

## Abstract

Myotonic Dystrophies type 1 (DM1) and type 2 (DM2) are complex multisystem diseases without disease-based therapies. These disorders are caused by the expansions of unstable CTG (DM1) and CCTG (DM2) repeats outside of the coding regions of the disease genes: *DMPK* in DM1 and *CNBP* in DM2. Multiple clinical and molecular studies provided a consensus for DM1 pathogenesis, showing that the molecular pathophysiology of DM1 is associated with the toxicity of RNA CUG repeats, which cause multiple disturbances in RNA metabolism in patients’ cells. As a result, splicing, translation, RNA stability and transcription of multiple genes are misregulated in DM1 cells. While mutant CCUG repeats are the main cause of DM2, additional factors might play a role in DM2 pathogenesis. This review describes current progress in the translation of mechanistic knowledge in DM1 and DM2 to clinical trials, with a focus on the development of disease-specific therapies for patients with adult forms of DM1 and congenital DM1 (CDM1).

## 1. Introduction: From the Molecular Advances to Pre-Clinical and Clinical *Studies in DM*

**Figure 1:**
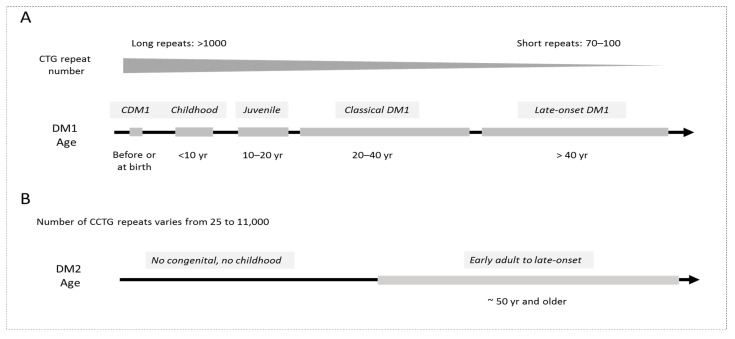
(**A**). Clinical forms of DM1 are shown. The longest CTG expansions are in CDM1 patients. Patients with short CTG repeats are affected later in life with mild symptoms. (**B**). DM2 is an adult disease with vary variable length of CCTG expansions.

Myotonic Dystrophies type 1 and type 2 are complex genetic diseases caused by unstable CTG expansions (from 37 to up to several thousand repeats) in the 3′UTR of the *DMPK* gene (DM1) and CCTG expansions in the first intron of the *CNBP* (also known as *ZNF9*) gene (DM2) [1,2]. Both diseases are characterized by a broad spectrum of multiple clinical symptoms, including defects in skeletal muscle, heart, brain and endocrine system [3,4,5,6]. There are five clinical forms of DM1, which include congenital DM1 (CDM1), childhood, juvenile, classical adult and late-onset DM1 (Figure 1). CDM1 is the most devastating form of DM1, which is associated with very long CTG expansions (>1000 repeats) affecting survival and development. These patients are characterized by extreme muscle weakness (hypotonia) and respiratory deficiency. CDM1 survivors display learning disability, motor delay and autistic disease symptoms during childhood, while in adulthood, they show symptoms of classical DM1 with myotonia and weakness. Juvenile DM1 is associated with behavioral features and cognitive involvement. Adult, classical DM1 is characterized by the development of myotonia, skeletal muscle weakness and wasting and progressive myopathy. In addition, patients develop cardiac conduction defects, cataracts, cognitive dysfunction, including attention, executive, memory and visuospatial defects, and predisposition to type 2 diabetes. Late-onset DM1 is associated with cataracts, mild weakness and myotonia after 40 years of age. 

DM2 is clinically similar to DM1; however, it has specific and distinct features. DM2 disease is characterized by the defects in skeletal muscle (muscle weakness, atrophy and myotonia); however, in DM2, mostly proximal muscles are affected, while in DM1, distal muscles are affected. Skeletal muscle pain is an important feature of the DM2 phenotype. DM2 patients also develop cardiac problems, predisposition to type 2 diabetes, cataracts and CNS abnormalities, including mild brain atrophy. There are no clinical forms in DM2 and no congenital and childhood disease, except of a few rare possible cases (reviewed in [6]). 

The lengths of CTG and CCTG repeat expansions are very variable. There is an approximate correlation between the length of CTG repeats and the severity of the symptoms in DM1, with the longest expansions in CDM1 patients and shortest expansions in very mild DM1 (Figure 1). The CTG expansions are also unstable in intergenerational transmissions and, as a result, the length of CTG expansions is increased in affected children (the phenomenon of genetic anticipation). Therefore, CDM1 patients with the longest expansions are affected at birth, while patients with short expansions (70–100 repeats) develop a very mild disease later in life. 

Although patients with DM2 have longer expansions than patients with DM1, the overall phenotype in DM2 is milder than in DM1. Correlation of the length of CCTG repeats with the disease severity in DM2 is problematic because patients might have very short or very long expansions and because DM2 symptoms could be mild. The disease onset in DM2 is typically in adulthood with very variable symptoms but the disease might progress after 50 years of age. 

Since both DM1 and DM2 are genetic diseases, the diagnosis of DM can be made by genetic testing, addressing the presence of CTG or CCTG repeats in the *DMPK* or *CNBP* genes using blood samples. The exact evaluation of the length of very long CCTG expansions might be difficult. Some reports suggest using a fluorescent in situ hybridization (FISH) assay detecting the CCTG repeats by hybridization with a CAGG probe combined with immunostaining with antibodies to MBNL1 (reviewed in [6]). However, this approach might have problems to distinguish DM1 and DM2 due to partial homology between CTG and CCTG repeats and because the sequestration of MBNL1 occurs in both diseases. 

Currently, there are no disease-specific therapies for both types of DM. There are symptomatic treatments for myotonia, pain and hypersomnia in these patients and monitoring of cardiac function, hypogonadism and insulin resistance [4,6]. 

It was shown that the expansions of CTG and CCTG repeats in DM1/2 cause diseases at the RNA levels due to toxicity of the accumulating mutant RNAs, containing expanded CUG and CCUG RNAs [7,8,9,10,11]. These molecular discoveries opened the door to initiate development of disease-specific treatments for DM. The use of patient-specific materials, such as muscle biopsies and DM1- and DM2-derived myoblast cell lines, helped to identify molecular therapeutic targets of DM1 and DM2. The generation of well-characterized mouse models [8,9] allowed researchers to test developing therapeutics for DM1. However, despite the large number of successful pre-clinical studies, the progress in the development in the clinical trials for DM1 is limited. Even less progress has been made in the development of therapeutic approaches for DM2. In this review, we will discuss the progress and challenges we face in the development of therapeutic clinical trials for DM1 and DM2 patients. 

## 2. Therapeutic Targets in DM1 and DM2

**Figure 2:**
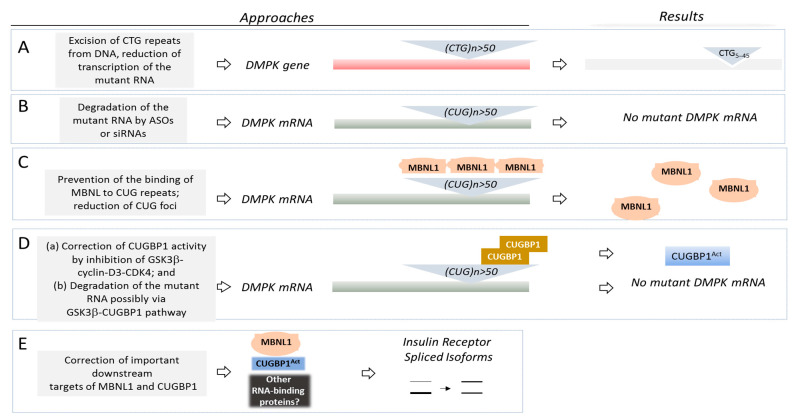
(**A**–**E**): The main therapeutic approaches for DM1 are shown. See text for details. *DMPK* gene and *DMPK* mRNA are shown as red and dark grey boxes. MBNL1 protein is shown in pink. Inactive CUGBP1 is shown in brown, while active CUGBP1 (CUGBP1^Act^) is shown in blue. Other RNA-binding proteins might participate in the regulation of MBNL1 and CUGBP1 targets in DM1.

The development of therapeutics for DM1 and DM2 requires precise knowledge of the molecular bases of these diseases. Since the molecular pathophysiologies of DM1/2 disorders are very complex, it is likely that combinatory drugs, affecting more than one target, might be used for the treatment of these diseases. In patients with DM1, the expanded CTG repeats within the 3′UTR of the *DMPK* gene lead to an accumulation of the mutant CUG-containing *DMPK* mRNA (which becomes toxic for the cells) (Figure 2). Since the mutant CUG-containing mRNA is very stable, it accumulates in the patients’ tissues in insoluble form (CUG foci) and in soluble, diffused form [10,11]. It has been found that several RNA binding proteins specifically recognize CUG repeats and these proteins are taken out from the pools, reducing their levels and/or changing their activities. Particularly, the CUG-containing mutant *DMPK* mRNA causes various toxic effects in the patients’ cells via interactions with families of MBNL (muscleblind-like) proteins and CUG-binding proteins, CUGBP (also known as CUGBP and ETR-like proteins or Elav-like proteins, CELF) [11,12,13,14,15,16,17]. These proteins regulate many activities in mRNAs at different levels, including splicing, translation, polyadenylation and RNA stability/decay. Thus, while the DM mechanistic studies focus on the splicing changes in DM1, it is important to note that RNA-binding proteins, affected by the mutant CUG repeats, have many other functions in addition to regulation of splicing. Therefore, the RNA homeostasis in DM1 is misregulated on several levels. Since the CUG-binding proteins’ function in various tissues is affected in DM, such as skeletal muscle, brain and heart, the CTG-CUG-CUGBP/MBNL pathways misregulate RNA metabolism in the patients’ tissues, causing multiple symptoms. In addition to CUGBP1 and MBNL families of RNA-binding proteins, other RNA-binding proteins are also affected by the mutant CUG repeats in DM1; however, their roles in DM1 pathophysiology are less investigated. 

While the mutant CUG repeats sequester MBNL proteins, reducing the activity of these proteins [16], the effect of the mutant CUG repeats on CUGBP1 is more complicated. In DM1, CUGBP1 is bound to the base of CUG hairpin [18], resulting in its stabilization and increased levels of CUGBP1 [19,20]. In addition, the pool of elevated CUGBP1 in DM1 cells consists of active CUGBP1 (phosphorylated at Ser302) and inactive CUGBP1 (un-phosphorylated at Ser302) [21]. These forms of CUGBP1 display different biological functions in DM1 cells. Given these findings, potential therapeutic approaches with a simple reduction in CUGBP1 would be insufficient to reduce the DM1 pathology because normalization of CUGBP1 activity is also critical. 

The pathological role of the accumulation of inactive CUGBP1 in DM1 cells was confirmed in pre-clinical studies [22,23] and in the recent CDM1 clinical trial [24]. It was shown that the accumulation of inactive CUGBP1 in the brains of CUGBP1-S302A knock in mice misregulated mRNA targets that are similar to those affected by the mutant *DMPK* mRNA in DMSXL mouse brains (mouse model with >1000 CTG repeats mimicking CDM1) [23]. In agreement, the reversion of inactive CUGBP1 into active CUGBP1 with the inhibitors GSK3β, tideglusib or TDZD-8 in the pre-clinical studies in DM1 mice (*HSA* model) and in DMSXL mice had a positive effect on CNS and neuro-muscular functions [22,23]. As we will discuss later, tideglusib also had a positive effect on the cognitive dysfunction and neuro-muscular defects in patients with CDM1 in a Phase II clinical trial [24]. Thus, the main mechanism of DM1 is a toxic RNA gain-of-function pathway, mediated by the accumulation of CUG repeats affecting RNA-binding proteins. 

It was suggested that the mutant RNAs in DM1 and in DM2 might also affect patients via repeat-associated non-AUG (RAN) translation, resulting in the accumulation of toxic polypeptides [25,26]. In addition, microRNA changes [27] and alterations in signaling pathways, directly or indirectly affected by CUG repeats, play a role in DM1 pathogenesis [11,28]. 

Toxic RNA gain-of-function mechanisms, identified in DM1 and DM2, have been described for other neurodegenerative diseases [29,30]. As an example, Fragile X-associated tremor/ataxia syndrome (FXTAS) is caused by the expansion of CGG repeats in the pre-mutation rate (55–200 repeats) within the 5′ UTR of the *FMR1* gene. In this disease, CGG repeats recruit heterogeneous nuclear ribonucleoprotein (hnRNP) A2/B1, which, in turn, brings to the repeat other RNA-binding proteins, such as CUGBP1, reducing their functions [31]. Overexpression of hnRNPA2/B1 and CUGBP1 rescues neurodegeneration in flies expressing CGG repeats. It has been suggested that RAN translation could also play a role in FXTAS [32]. In addition, CGG aggregates might recruit several RNA-binding proteins, including MBNL1 [33]. There is also possible mechanistic similarity in some neurodegenerative diseases due to a connection with cellular stress [32]. In Spinocerebellar ataxia type 8 (SCA8), bi-directional expression of CAG-CTG expansion results in CUG-containing RNA, which misregulates MBNL1 and CUGBP1 [34]. The RNA gain-of-function mechanism was suggested for Fuchs endothelial corneal dystrophy (FECD) [35]. Amyotrophic lateral sclerosis and frontotemporal dementia (C9ORF72 ALS/FTD), caused by non-coding GGGGCC repeats [36,37], might also be associated with the toxic RNA gain-of-function and RAN translation [29,30]. 

Based on the molecular mechanisms of DM1, multiple approaches were proposed to test DM1 therapeutics in preclinical studies, reviewed in detail in several comprehensive papers [28,38,39]. One of the main approaches is the deletion of CTG expansion from the genomic DNA (Figure 2A). Although this approach is still far from the clinical studies, it is very important because it might remove the DM1 mutation at the level of genomic DNA. The approaches focused on the removal of mutant *DMPK* mRNA and correction of two major RNA-binding proteins, CUGBP1 and MBNL1, involved in DM1, or their critical downstream targets are tested in the clinical studies; therefore, they might be closer to the transition to clinic (Figure 2B–E). 

### 2.1. Excision of the DM1 Mutation from the Genomic DNA

The first logical therapeutic approach suggested a deletion of the expanded CTG repeats from the mutant *DMPK* gene (Figure 2A). This approach is based on the excision of the portion of the *DMPK* gene containing CTG repeats using the clustered, regularly interspaced, short palindromic repeats (CRISPR)-Cas system. The advantage of this approach is that the DM1 mutation could be eliminated from the genomic DNA, preventing all toxic downstream effects caused by CTG expansion. This approach was tested by different groups using immortalized myoblasts derived from muscle biopsies of patients with DM1, DM1-specific-induced pluripotent stem cells (iPSs), myogenic cells derived from the DM1-iPS cells [40,41,42,43] and myoblasts derived from DM1 mice expressing 500 CUG repeats (DM500) [40]. The deletion of mutant CTG repeats in cultured cells reduced the number of CUG foci, corrected delayed DM1 myoblast differentiation and rescued mis-splicing events [40,41,42,43]. It has been found that a single intramuscular injection of recombinant adenoviral vectors, expressing Cas9 nuclease and two single-guided RNAs in DMSXL mice, deleted CTG repeats in muscle [42]. As a result, the toxic CUG-containing aggregates were reduced. Thus, the CRISPR/Cas system could be used to remove CTG repeats from the genomic DNA in patients with DM1 and CDM1. This approach could also be used to reduce the transcription of the mutant CUG-containing RNA [43,44]. However, there are several potential obstacles in the CRISPR/Cas approach that remain to be solved. First, CTG repeats form a secondary structure that might interfere with the specific recognition and the binding of the CRISPR-Cas components (reviewed in refs. [43,45]). Second, this approach requires a viral delivery of the components in the CRISPR-Cas system. Since DM1 is a multisystem disease, the components in the CRISPR/Cas system have to be delivered in all affected tissues. Third, there might be some problems with the preexisting immunity and immune response to the delivery system. Fourth, there might be possible off-target mutations. The development of this therapeutic approach in DM1 continues in preclinical studies. At this stage, the genetic editing of a DM1 mutation using the CRISPR/Cas approach needs additional work.

### 2.2. Degradation of the Mutant DMPK mRNA

The removal of the mutant *DMPK* mRNA from the patients’ tissues using AONs and siRNAs was developed by several groups as one of the logical therapeutic approaches to treat DM1 (Figure 2B). The degradation of the mutant *DMPK* mRNA should correct all downstream toxic pathways, including misregulation of RNA-binding proteins, such as CUGBP1 and MBNL1, as well as other RNA-binding proteins targeted by CUG repeats. Several methods to degrade the mutant *DMPK* mRNA were proposed, including antisense oligonucleotides (AONs) with different modifications, complimentary to the mutant gene and small interfering RNAs (si-RNAs) [46,47,48,49]. The application of AONs and siRNAs was successful in preclinical studies and allowed researchers to reduce the mutant *DMPK* mRNA, decreasing myotonia and correcting DM1-associated mis-splicing events [46,47,48,49]. The preclinical data using *DMPK*-specific AON led to the first Phase ½ clinical trial, based on the DM1-specific pathogenesis, in Ionis Biopharmaceutical (Table 1). 

Despite successful testing of AON targeting mutant CUG RNA in pre-clinical studies [47], the use of *DMPK*-specific AON faced with difficulties for the effective delivery to the affected tissues (such as skeletal muscle) in human patients with DM1 [50]. Currently, new approaches, improving AON delivery are being suggested. One of them is application of the peptide- or other ligand-conjugated oligonucleotides, which might improve AON delivery in skeletal and cardiac muscles [51,52,53]. 

Recent reports showed that the delivery of the *DMPK*-specific AON in DMSXL mice via intracerebroventricular injections reverses behavioral defects [54]. The development of this approach might lead to clinical studies, addressing correction of CNS defects in DM1. Lack of tissue selectivity and low membrane permeability in AONs and small interfering RNAs could be improved by using AONs conjugated with antibodies, forming an antibody–drug conjugate (ADC) [55]. This approach was used by Avidity Biosciences, Inc, which is applying a conjugate of specific antibodies that bind to the transferrin receptor 1 and a small interfering RNA, targeting *DMPK* mRNA (AOC 1001) in the Phase ½ MARINA trial for the treatment of patients with adult DM1 (Table 1). This study is addressing safety and tolerability of AOC 1001 administered intravenously [56]. Regarding side effects of AON application, clinical trials for other diseases using AONs therapeutics led to thrombocytopenia, hepatic toxicity and immune response [45]. One of the limitations of AONs use is that they might target both normal and mutant RNAs [57]. In summary, additional studies are needed to develop successful AONs or siRNAs targeting the mutant *DMPK* mRNA in DM1 clinical trials. 

### 2.3. Correction of Activities of RNA-Binding Proteins as the Therapeutic Approach for DM1 and CDM1

While the developments of the approaches targeting the DM1 mutations and the mutant *DMPK* mRNA using AONs are in progress, DM1 pathology could be corrected via normalization of RNA-binding proteins, CUGBP1 and MBNL1. It was expected that correction of at least one RNA-binding protein, affected in DM1, might reduce some symptoms in DM1 patients. It is also possible that the use of the combinatory drugs correcting both MBNL1 and CUGBP1 could improve the DM1 pathology even more. In the case of the correction of CUGBP1 activity in DM1, the normalization of CUGBP1 activity in DM1 also has a positive effect on the degradation of the mutant CUG-containing RNA (Table 1). Thus, the decay of the mutant CUG repeats could be easier to achieve using small-molecule drugs as therapeutics for the correction of biological activities in CUGBP1. Disruption of MBNL1 binding to the mutant CUG repeats and the reduction in the number of CUG foci might also improve the degradation of the mutant RNA. 

#### 2.3.1. Tideglusib Treatments Corrected CNS and Muscle Defects in a Phase II Clinical Trial in Patients with CDM1

Several reports suggested that inhibitors of GSK3β might be considered for treatments in DM1 patients. First, GSK3β kinase is increased in DM1 patients [22]. Second, cyclin D3 is a substrate of GSK3β and cyclin D3-CDK4 regulates CUGBP1 activity by phosphorylation at Ser302 [21]. It has been shown that the increase in GSK3β in DM1 affects the cyclin D3-CUGBP1 pathway, contributing to the DM1 muscle phenotype (myotonia, atrophy, muscle weakness and myopathy) in DM1 mice [22,23] and muscle weakness, myopathy and anxiety in DMSXL mice [23]. Based on the activation of this toxic pathway in DM1 patients, AMO Pharma performed a Phase IIb clinical trial targeting GSK3β and, respectively, the toxic GSK3β-cyclin D3-CUGBP1 pathway in adult patients with congenital and childhood-onset DM1 [24] (Table 1). This study addressed the safety and tolerability of tideglusib. It was shown that tideglusib is generally safe and well tolerated. The overall results of the Phase II clinical trial testing tideglusib were promising with improvements in CNS and neuromuscular symptoms in CDM1 patients. Although the increase in alanine aminotransferase as a side effect in some participants was observed, it was reversible. One of the challenges in the analysis of the clinical studies in DM1 is that the DM1 phenotype is complex and is variable from patient to patient. For example, all participants in the AMO Pharma study (n = 16) showed communication difficulties; however, limitations with mobility, difficulty thinking, fatigue, problems with hands and arms, emotional issues, myotonia and vision problems were observed in the majority of patients [24]. Almost half of the group showed sleepiness features, gastrointestinal issues, pain, choking, swallowing problems and breathing difficulties. Inability to perform activities, decreased social satisfaction and decreased social performance were observed in about one-third of the patients in the described group. As has been shown, the treatments with tideglusib (normalizing the GSK3β-cyclin D3-CUGBP1 pathway) improved social performance (fatigue, sleepiness and gastrointestinal issues) in most of the CDM1 patients treated with either low or high doses of tideglusib. Other symptoms of the CNS and neuromuscular system, such as myotonia, communication difficulties, choking or swallowing, decreased social satisfaction, pain, difficulty thinking and problems with hands and arms were improved in approximately half of the treated patients. Some improvements in the limitations with mobility, emotional issues, inabilities to do activities, problems with vision and breathing difficulties were also observed in the treated patients [24]. 

The successful development of the AMO Pharma trial was supported by preclinical findings in the mouse models for DM1 and in DMSXL mice with long CTG expansions, in which the treatments with tideglusib or tideglusib analogues led to significant improvements in skeletal muscle symptoms, such as muscle weakness, atrophy, skeletal muscle histopathology and myotonia [22,23]. Behavioral defects were also improved in the DMSXL mice treated prenatally with tideglusib [23]. The promising results of the Phase II clinical trial at AMO Pharma prompted further development of the study to a Phase II/III clinical trial in children and adolescents with CDM1 (6–16 years of age) [58]. This study will analyze muscle and CNS symptoms in patients treated with tideglusib. While the AMO-02 clinical trial is focusing on patients with CDM1, it is important to investigate whether tideglusib is effective in patients with classic, adult forms of DM1. The pre-clinical studies suggest that tideglusib might also be beneficial in patients with adult forms of DM1 [22,23]. Additional studies are needed to address if the mutant CUG repeats are degraded in the CDM1 patients treated with tideglusib.

#### 2.3.2. Small Molecules as Therapeutics Correcting MBNL1 and CUGBP1 in DM1

It has been suggested that the increased stability of the mutant CUG-containing mRNA might be due to binding of MBNL1 to the mutant CUG repeats. Therefore, many studies have been focused on the identification of small molecules and other approaches that might disrupt binding of MBNL1 to the mutant CUG repeats and might reduce the number of CUG foci, improving splicing of mRNAs, regulated by MBNL1 (reviewed in references [59,60,61,62,63]). These small molecules have been identified using the screening of the compound libraries or synthesized by special design. Main criteria to evaluate the efficacy of the identified small molecules included prevention of MBNL1 binding to the expanded CUG repeats, reduction in CUG foci and correction of splicing targets, known to be misregulated in DM1 using cell culture lines from patients with DM1 or DM1 mice. Using these parameters, various small molecules correcting MBNL1 activity and improving splicing were identified and their number is growing fast [59,60,61,62,63,64,65,66,67,68,69,70,71,72,73]. The list of small molecules that improve MBNL1 activity includes anti-infective agents (such as pentamidine, furamidine and erythromycin) [64,65,66], compounds affecting microtubules [68] and small molecules increasing MBNL1 levels (such as the anti-autophagic drugs and inhibitors of HDAC) [67,71]. The identification of these small molecules suggests the possibility of MBNL1 correction in DM1. Currently most of the studies of the candidate small molecules correcting MBNL1 are at the pre-clinical stage. These molecules mainly correct splicing and reduce the number of CUG foci in DM1 models. Some of them, such as erythromycin and furamidine, reduce myotonia in DM1 mice [65,66]. Since erythromycin is relatively safe, it is being used in the Phase II clinical trial for adult patients with DM1 [74]. 

Additional direction of search for drug small molecules for DM1 included screening of the libraries of the kinase inhibitors based on the reduction in CUG foci [28,63,75,76]. In this regard, it was found that the inhibitor of PKC kinase Ro 31-8220 reduces CUG foci in DM1 cells and increases the cytoplasmic levels of MBNL1 [75]. Interestingly, this molecule also corrects CUGBP1 levels. It would be important to examine if small molecules reducing CUGBP1 levels also rescue CUGBP1 activity since normalization of CUGBP1 should include correction of CUGBP1 levels as well as rescue of CUGBP1 activity via correction of the GSK3β-cyclin D3-CDK4 signaling pathway. Other small molecules, inhibitors of kinases, which improve MBNL1 and CUGBP1 in DM1 cells and reduce the accumulation of the mutant CUG-containing transcripts, were identified [28,76].

Despite the large number of candidate small molecules disrupting MBNL1 binding to CUG repeats, more studies are needed to examine the therapeutic effects of these potential drugs in vivo. Monitoring the drugs’ efficacy based on the number of CUG foci might have some difficulties because the number of CUG foci and their brightness is variable from cell to cell. The measurements of splicing changes as an outcome of the drug effect also have some difficulties. The main issue is variability in splicing changes in different patients, including very small splicing alterations for some genes. Thus, confirmation of the benefits of small molecules should include the analysis of the drug effect on DM1 phenotype in vivo. In addition, selection of a few genes whose splicing patterns are reproducible in many patients with DM1 might be helpful in the evaluation of MBNL1 activity.

#### 2.3.3. Downstream Targets of the Main RNA-Binding Proteins, Misregulated in DM1

Recent studies showed that therapeutics that correct downstream targets of MBNL1 and CUGBP1 could also be used for DM1 therapy. Among the first identified splicing targets of CUGBP1 and MBNL1, misregulated in DM1, is insulin receptor, IR [77]. This target seems to be involved in both DM1 and DM2 pathogeneses because both DM1 and DM2 are characterized by insulin resistance and predisposition to type 2 diabetes (T2D) [3,4,5,6]. It was shown that an anti-diabetic drug, metformin, corrects abnormal IR splicing in DM1 mesodermal precursor cells (MPCs) and in DM1 myoblasts ([78] and reviewed in [79]). Interestingly, in addition to IR, metformin also improves splicing of other genes, including *TNNT2 and Clcn1* [78]. It has been found that the effect of metformin did not involve MBNL1 or CUGBP1 but was associated with changes in RNA-binding protein RBM3, which also regulates splicing. The positive effect of metformin on the RBM3 was associated with AMP-activated protein kinase (AMPK) since the activator of AMPK, AICAR (5-Aminoimidazole-4-carboxamide ribonucleotide), regulated the RBM3 levels [78]. Since metformin is relatively safe, it was used to treat DM1 patients (18–60 years of age) in a Phase II clinical trial [80]. Metformin significantly increased mobility in DM1 patients, based on the results of a 6 min walk test and on the improvement in gait ability. However, treated patients did not show changes in myotonia or muscle weakness. Side effects of metformin included some gastrointestinal problems. Splicing changes were measured in the blood samples from the metformin-treated DM1 patients; however, sensitivity of the splice isoforms detection was too low to make conclusions if the IR splicing was improved in DM1 patients. Regardless of the exact mechanism of action, metformin treatment is used in a currently active Phase III clinical trial (Table 1). Based on the knowledge that metformin might act as anti-aging drug [81], it might be beneficial in DM1, at least as a drug supplementing other therapeutics. Since metformin is known to activate AMPK signaling, several studies analyzed the involvement of AMPK signaling in DM1 [82,83]. It was found that AICAR improves muscle histopathology and reduces myotonia in *HSA^LR^* mice, correcting splicing of *Chch1* [82,83]. AICAR also had a positive effect on the muscle force in DM1 mice [82]. In addition, AICAR treatments reduce the number of CUG foci in muscle of *HSA^LR^* muscle [82,83]. It was shown that mTORC1 signaling was also deregulated in *HSA^LR^* mice [82]. An mTORC1 inhibitor, rapamycin, improved muscle function (myotonia and strength) in *HSA^LR^* mice but without splicing changes.

**Figure 3:**
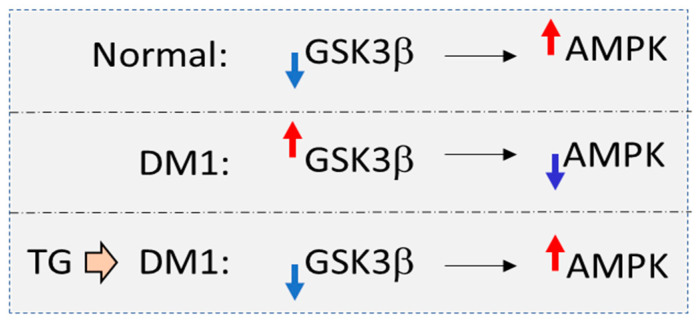
Possible involvement of GSK3β kinase in the regulation of AMPK in DM1. In normal cells, high levels of active AMPK are maintained by the low levels of GSK3β activity. However, in DM1 cells, GSK3β is elevated. Since AMPK is a substrate of GSK3β, the increase in GSK3β may lead to a reduction in AMPK in DM1 cells. It is expected that the correction of GSK3β activity with tideglusib (TG) in DM1 cells might normalize levels of AMPK.

It has been found that the age-associated drug Resveratrol (RSV) (which activates AMPK signaling) also had a positive effect on splicing in *HSA^LR^* mice [83]. The mechanism by which AMPK signaling is misregulated in DM1 remains to be investigated. One possibility is that control of AMPK, regulated by CaMKII kinase, is altered in DM1 mice due to a reduction in CAMKII because of misregulation of CAMK splicing [82]. Another possibility is that AMPK is misregulated in DM1 mice due to an increase in GSK3β. Since AMPK is one of the substrates of GSK3β [84], an increase in GSK3β in DM1 [22] might have a negative effect on AMPK (Figure 3). Inhibition of GSK3β with small-molecule inhibitors, such as tideglusib, might activate AMPK, correcting insulin resistance. While the mechanisms for the correction of insulin resistance in DM1 should be further investigated, metformin might have, at least in part, some beneficial effects in DM1.

## 3. Therapeutic Studies in DM2

Development of the therapeutic approaches for DM2 is progressing much slower than development of a therapy for DM1. This is associated with relatively late discovery of the DM2 mutation (2001) [2] vs DM1 (1992) [1]. There are also some questions whether the mutant CCUG repeats cause pathogenesis by the same mechanisms as CUG repeats in DM1. Despite similarities in the clinical phenotypes in DM1 and DM2, there are specific clinical features in DM2, including defects in different muscles and lack of the congenital form of DM2. While both diseases are caused by unstable expansions, the CTG (DM1) and CCTG (DM2) expansions are located within the genes, encoding proteins with unrelated functions. The locations of the DM1 and DM2 expansions within the corresponding genes are also different. In DM1, CTG expansion is in the 3′ UTR of the *DMPK* gene and this expansion affects DM1 cells as a part of the mutant *DMPK* mRNA. However, in DM2, expanded CCTG repeats are located within the intron 1 of *CNBP* gene. Under normal conditions, the introns are usually quickly degraded after splicing; however, the splicing of the mutant intron 1 in *CNBP* pre-mRNA is reduced [85]. Thus, the mutant CCTG repeats negatively affect DM2 cells as a part of the mutant *CNBP* pre-mRNA. These molecular similarities and differences suggest that while some therapeutic approaches might be similar in both diseases, there might be disease-specific therapeutic targets. 

**Figure 4:**
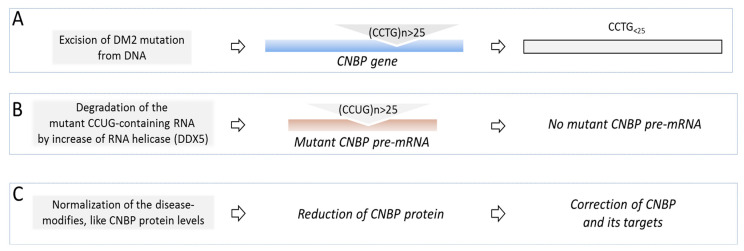
(**A**–**C**): The models of the proposed therapeutic approaches for DM2 (see text). Mutant *CNBP* gene and *CNBP* mRNA are shown as blue and brown boxes.

Another issue with the development of therapy for DM2 is that there is a delay with the development of mouse models for DM2, which are needed for drug screening. The DM2 mouse model, containing 121 CCTG repeats within the intron 1 of the human skeletal actin gene, was generated; however, the preliminary analysis of this model showed that these mice develop DM2 symptoms without splicing defects [86]. Interestingly, a recent study showed that MBNL1 splicing targets are altered in DM1-iPS-derived cardiomyocytes, but not in DM2 cells [87]. Thus, additional studies on DM2 mouse models with 121 CCTG repeats are needed. Further, examination of new mouse models with longer CCTG expansions would be important. Similar to CUG repeats in DM1, the CCTG expansion could be deleted from the genomic DNA with the CRISPR/Cas system (Figure 4). However, to our knowledge, there are no reports showing if this approach for DM2 is under development.

Degradation of the mutant CCUG-containing RNA with specific AONs in DM2 might have difficulties. If AONs target *CNBP* pre-mRNA, this would also reduce *CNBP* mRNA levels, leading to a reduction in CNBP protein. Since CNBP has essential functions in normal cells (reviewed in [88]), the reduction in CNBP should be avoided. The mutant CCUG RNA could be also degraded by the normalization of the Dead-box 5 (DDX5) RNA-helicase p68 [89] (Figure 4). Since p68 is reduced in DM1 and in DM2, potential normalization of p68 could improve degradation of the mutant CUG- and CCUG-containing RNAs. However, to our knowledge, no approaches to improve p68 expression in DM1 or DM2 have been developed. 

A search for the drugs that reduce MBNL1 binding to CCUG repeats by the screening of the compound libraries identified various small molecules that could reduce the number of CCUG foci and correct mis-splicing associated with MBNL1 sequestration in DM2 cells [59,90,91,92,93,94,95]. These compounds were tested in pre-clinical studies mainly in DM2 cell lines and in the mutant CCUG-expressing flies addressing their effects on the splicing of the MBNL1 targets and reduction in CCUG foci. It would be important to examine the effect of these molecules in vivo using DM2 mouse models. Importantly, some identified compounds, which inhibit TGFβ-activin signaling, rescued muscle degeneration in DM2 flies [91]. Although MBNL1 is sequestered by the mutant CCUG repeats, the role of this event in DM2 pathogenesis is not fully understood. Since the length of CCTG repeat expansions in DM2 is bigger than the length of CUG repeats in DM1, they should deplete bigger amounts of MBNL1 protein, making the DM2 phenotype more severe than in DM1. However, DM2 is milder than DM1, suggesting that sequestration of MBNL1 might be only partially involved in DM2 pathogenesis and other factors (such as RNA-binding protein rbFOX1 [96]) might play a role. There is also a possibility to correct DM2 by improving some potential modifying factors, associated with DM2, such as CNBP. CNBP is a DNA- and RNA-binding protein that regulates gene expression at the levels of transcription and translation [88]. It plays a significant role in development, immune system and tumorigenesis. 

Several reports suggested that CNBP protein is reduced in DM2 [97,98,99], although some studies found that CNBP expression is not altered in DM2 [100,101,102]. However, recent publications showed that the processing of the mutant *CNBP* mRNA is affected in DM2 [85] and that the levels of CNBP are reduced in myoblasts from patients with DM2 [103]. Since CNBP regulates many mRNAs [104], a reduction in CNBP levels or its activity might contribute to the disruption of RNA metabolism in DM2 patients. Several mouse models were generated in which *Cnbp* was deleted [105,106,107]. Deletion of *Cnbp* causes myotonia, cardiac defects and muscle histopathology [105]. A mouse model, in which *Cnbp* gene was disrupted, developed late muscle atrophy and weakness [106]. Deletion of *Cnbp* could also contribute to the defects in immune system [107]. Therefore, a reduction in CNBP might be associated with at least some symptoms in DM2. It has been shown that CNBP stability could be regulated by phosphorylation by AMPK kinase (reviewed in [88]). Small molecules increasing CNBP stability might normalize CNBP levels, contributing to the correction of DM2 pathogenesis. Thus, the search for the therapeutic targets and potential drugs in DM2 is at the initial stages and appropriate mouse models should be generated to test the identified candidate drugs for DM2. 

## 4. Conclusions and Further Studies

**Myotonic Dystrophy 1.** Clinical trials Phases II-III are testing three potential candidate drugs that might be beneficial in CDM1 and in DM1. They include a small-molecule inhibitor of GSK3β, tideglusib, metformin and erythromycin. 

**Tideglusib** showed promising improvement in the cognitive dysfunction and neuro-muscular symptoms in patients with CDM1 in a Phase II clinical trial [24]. Currently, this drug is being tested in a Phase III clinical trial. The results of this trial will be critical to determine if tideglusib could be used for the treatment of CDM1 alone or in combination with other drugs. It would be also important to determine the efficacy of tideglusib in the clinical trials for patients with adult forms of DM1. The treatments for DM1 with tideglusib are directly connected to the core mechanisms of DM1, in which the toxic CUG-containing RNA affects RNA-binding protein, CUGBP1, by converting active CUGBP1 into CUGBP1 repressor. Since activity of CUGBP1 is controlled by GSK3β, correction of GSK3β in CDM1 or DM1 models restores CUGBP1 activity and improves muscle (myotonia, weakness, atrophy, myopathy) and CNS (anxiety) phenotypes. Based on the clinical trial Phase II, tideglusib partially corrected myotonia, fatigue and cognition defects in patients with CDM1 [24]. In addition to the correction of CUGBP1 activity, tideglusib reduces the mutant CUG RNA in a DM1 model [23]. It remains to be determined whether tideglusib converts CUGBP1 activity in patients with DM1 and whether the mutant *DMPK* mRNA is degraded in the treated patients. There should be no difficulties monitoring tideglusib efficacy in clinical trials. 

**Metformin:** Based on the results of the Phase II clinical trial [80], it is expected that muscle performance might be improved in DM1 patients treated with metformin. It is possible that other parameters in the DM1 phenotype might also be improved. The use of metformin is based on the correction of the specific symptom in DM1 (insulin resistance) and on the correction of the main downstream splicing target, mis-regulated in DM1, IR. However, it is still unclear whether metformin corrects IR splicing in the treated patients and whether it has additional positive effects besides mobility and gait improvement. The correction of AMPK signaling, associated with metformin, in DM1 is also an exciting approach.

**Erythromycin** might reduce myotonia and possibly other symptoms in DM1 in a clinical trial Phase II. This drug restores splicing and the MBNL1 activity in the mouse model. Thus, splicing biomarkers could be used to monitor the effect of erythromycin in treated patients with DM1. 

**Myotonic Dystrophy 2:** The DM2 mechanistic studies and therapeutic approaches are still in development. New data showing that DM2 could be caused by very short expansions (around 25 CCTG repeats) [108] further complicate the understanding of the molecular mechanism of DM2 associated with the toxicity of CCUG repeats. Thus, a better understanding of DM2 pathogenesis and the development of the in vivo mouse models will provide a background for the generation of candidate drugs for DM2. 

It remains to be determined if the correction of the additional molecular players in DM1 and in DM2, such as microRNAs [27], and the reduction in the accumulation of abnormal peptides synthesized due to RAN translation [25,26] would be beneficial in clinical trials for patients with these diseases.

## Figures and Tables

**Table 1:** Examples of clinical trials for DM1 which are based on correction of mechanisms, causing DM1 pathology (see text for details). AOC is antibody-oligonucleotide conjugate.

Targeted Mechanism of DM1	Company	Phase I	Phase II	Outcome of Phase II	Phase III
Correction of RNA-binding protein CUGBP1 and degradation of the mutant RNA by small molecule GSK3 inhibitor tideglusib	AMO Pharma	Drug safety is known	Phase 2 completed	Reduction of CNS and muscle defects	Active
Correction of splicing of Insulin Receptor and other splicing events by metformin	Tor Vergata	Drug safety is known	Phase 2a completed	Mobility and gait improvement	Active
Correction of MBNL1 activity, reduction of CUG foci, reduction of myotonia by erythromycin	Osaka University Hospital	Drug safety is known	Active		
Degradation of the mutant *DMPK* mRNA by AON	Ionis	Phase ½ completed	Phase ½ completed	Poor penetration into skeletal muscle	
Degradation of the mutant *DMPK* mRNA by AOC	Avidis	Phase ½ in progress			

## Data Availability

Not applicable.
